# *Genus_Ruminococcus* and *order_Burkholderiales* affect osteoporosis by regulating the microbiota-gut-bone axis

**DOI:** 10.3389/fmicb.2024.1373013

**Published:** 2024-05-21

**Authors:** Ning Li, Haiyang Wang, Huan Pei, Yueying Wu, Lei Li, Yu Ren, Si Wang, Yuan Ma, Miao Luo, Jiali Yuan, Lvyu Li, Dongdong Qin

**Affiliations:** ^1^First Clinical Medical College, Yunnan University of Chinese Medicine, Kunming, China; ^2^Key Laboratory of Integrated Chinese and Western Medicine for Chronic Disease Prevention and Control, Yunnan University of Chinese Medicine, Yunnan Province, Kunming, China; ^3^The Third People’s Hospital of Yunnan Province, Kunming, China; ^4^Kunming Municipal Hospital of Traditional Chinese Medicine, The Third Affiliated Hospital of Yunnan University of Chinese Medicine, Kunming, China; ^5^Key Laboratory of Traditional Chinese Medicine for Prevention and Treatment of Neuropsychiatric Diseases, Yunnan University of Chinese Medicine, Kunming, China

**Keywords:** osteoporosis, Mendelian randomization, 16S rDNA, g_*Ruminococcus*, o_*Burkjolderiales*, microbiota-gut-bone axis, animal model validation

## Abstract

**Background:**

This study aimed to clarify the relationship between the gut microbiota and osteoporosis combining Mendelian randomization (MR) analysis with animal experiments.

**Methods:**

We conducted an analysis on the relationship between differential bacteria and osteoporosis using open-access genome-wide association study (GWAS) data on gut microbe and osteoporosis obtained from public databases. The analysis was performed using two-sample MR analysis, and the causal relationship was examined through inverse variance weighting (IVW), MR Egger, weighted median, and weighted mode methods. Bilateral oophorectomy was employed to replicate the mouse osteoporosis model, which was assessed by micro computed tomography (CT), pathological tests, and bone transformation indexes. Additionally, 16S rDNA sequencing was conducted on fecal samples, while SIgA and indexes of IL-6, IL-1β, and TNF-α inflammatory factors were examined in colon samples. Through immunofluorescence and histopathology, expression levels of tight junction proteins, such as claudin-1, ZO-1, and occludin, were assessed, and conduct correlation analysis on differential bacteria and related environmental factors were performed.

**Results:**

A positive correlation was observed between *g_Ruminococcus1* and the risk of osteoporosis, while *O_Burkholderiales* showed a negative correlation with the risk of osteoporosis. Furthermore, there was no evidence of heterogeneity or pleiotropy. The successful replication of the mouse osteoporosis model was assessed, and it was found that the abundance of the *O_Burkholderiales* was significantly reduced, while the abundance of *g_Ruminococcus* was significantly increased in the ovariectomized (OVX)-mice. The intestinal SIgA level of OVX mice decreased, the expression level of inflammatory factors increased, barrier damage occurred, and the content of LPS in the colon and serum significantly increased. The abundance level of *O_Burkholderiales* is strongly positively correlated with bone formation factors, gut barrier indicators, bone density, bone volume fraction, and trabecular bone quantity, whereas it was strongly negatively correlated with bone resorption factors and intestinal inflammatory factors, The abundance level of *g_Ruminococcus* shows a strong negative correlation with bone formation factors, gut barrier indicators, and bone volume fraction, and a strong positive correlation with bone resorption factors and intestinal inflammatory factors.

**Conclusion:**

*O_Burkholderiales* and *g_Ruminococcus* may regulate the development of osteoporosis through the microbiota-gut-bone axis.

## 1 Introduction

As a chronic degenerative bone disease, osteoporosis has become an important health problem worldwide. The incidence of osteoporosis has increased dramatically recently, causing great harm to millions of people worldwide (Domazetovic et al., 2017; Yu and Xia, 2019; Salari et al., 2021; Willers et al., 2022). The etiology and pathogenesis of osteoporosis are complex, involving the stimulation of various hormones such as estrogen and parathyroid hormone as well as environmental and genetic factors (Marini et al., 2016).

The microecology of the gut is the most important and complex microecosystem in the human body, playing a crucial role in digestion, nutrition, metabolism immunity, etc. The influence of gut microorganisms and their metabolites on bone health has received widespread attention in recent years. Intestinal flora plays a key role in the development of osteoporosis, and targeting intestinal flora can delay the onset of osteoporosis in different ways, such as bacterial colonization, probiotic supplementation, and exercise intervention (Inchingolo et al., 2023), which has become a new target for osteoporosis diagnosis and treatment (Yuan et al., 2022). However, the mechanism of action between gut microbiota and osteoporosis is complex and intricate. For example, *Lactobacilli* can alleviate primary and secondary osteoporosis (Liu et al., 2020; Lee et al., 2021; Rastogi and Singh, 2022; Guo et al., 2023). However, in a systematic evaluation of the results of microbial gene sequencing in the gut of osteoporosis patients from several countries and regions of the world, it was found that the relative abundance of *Lactobacilli* was higher in the gut of patients with osteoporosis compared to the healthy controls (Huang et al., 2022). Additionally, the abundance of *Lactobacillus* in the gut microbiota of patients with diabetes and osteoporosis is significantly higher compared to healthy controls and is accompanied by gut barrier dysfunction and bacterial translocation (Knudsen et al., 2021). While the abundance of gut microorganisms may not fully indicate the role of flora in disease, the variability of results between basic and clinical studies indicates the need for a more scientific approach to validate the link between the gut microbiota and osteoporosis.

Due to the lack of evidence from randomized controlled trials, it is unclear whether there is a clear causal relationship between the gut microbiota and osteoporosis. Randomized controlled trials are the gold standard for inferring causality in medical research, and confounding bias can be minimized by randomized grouping. However, large-sample controlled trials have difficulties such as high costs and long trial periods. Therefore, to prove whether there is a causal relationship between observed correlations, a Mendelian randomization (MR) analysis can be performed (Evangelou et al., 2018). MR simulates the process of random grouping by using randomly distributed single nucleotide polymorphisms (SNPs) in genetic data as instrumental variables (IVs) (Smith and Ebrahim, 2003; Didelez and Sheehan, 2007; Davey and Hemani, 2014). According to Mendel’s second law, alleles are randomly assigned to individuals and are fixed at the time of egg fertilization, and the use of MR circumvents the effects of reverse causality and confounding environmental factors inherent in traditional epidemiological methods. To further investigate the connection between the gut microbiota and osteoporosis, we conducted animal experiments simultaneously utilizing the OVX replicated mouse model of bone loss. Additionally, we analyzed the variations in gut barrier indicators and intestinal inflammatory factors in mice experiencing bone loss, aiming to establish the correlation between specific bacteria and the gut-bone relationship, thereby confirming the impact of the microbiota-gut-bone axis on osteoporosis.

## 2 Materials and methods

### 2.1 MR analysis

**Figure 1:**
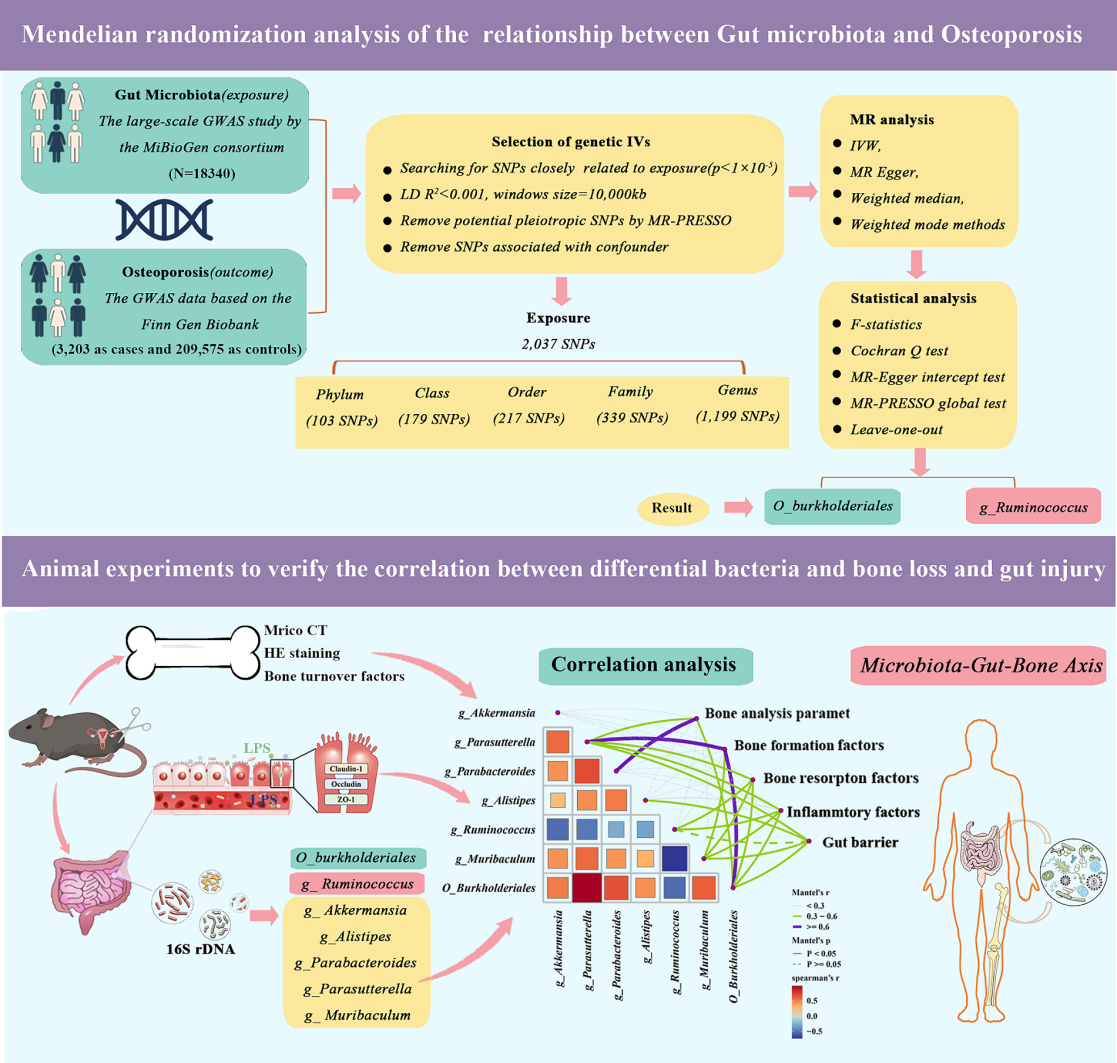
Validation of Mendelian randomization analysis and 16S rDNA sequencing reveals that *genus_Ruminococcus* and *order_Burkholderiales* affect osteoporosis by regulating the microbiota-gut-bone axis.

MR analysis requires the fulfillment of three essential assumptions, namely: (1) the SNPs used as instrumental variables should have a strong association with the study’s risk factors (correlation hypothesis); (2) the genetic variants used should not be linked to potential confounders (independence hypothesis); and (3) the genetic variants should only affect the risk of an outcome through the risk factors, and not through other pathways (exclusion restriction hypothesis). Our study relies on genome-wide association study (GWAS) summary-level data from the IEU Open GWAS project, which is run by the University of Bristol’s MRC Integrated Epidemiology Unit (IEU). In this project, GWAS data is collected and analyzed from a variety of sources, such as the UK Biobank, FinnGen Biobank, and published articles. As the data used in this study was publicly accessible, anonymized, and de-identified, no ethical review board approval was required. Figure 1 illustrates an overview of the research design of our study.

#### 2.1.1 Data sources

The pooled statistics on the gut microbiota were acquired from a comprehensive GWAS conducted by the MiBioGen consortium, encompassing a substantial sample size of 18,340 individuals belonging to European ethnic minority groups across 11 countries. The dataset comprised a total of 122,110 variant loci. The MiBioGen consortium’s official website^1^ provides further details on this study (Kurilshikov et al., 2021). Based on the GWAS, we screened IVs for gut bacterial taxa at five levels (phylum, class, order, family, and genus), excluding 15 bacterial taxa that did not have species-specific names, and the GWAS data for outcomes were selected for osteoporosis (3,203 as cases and 209,575 as controls) from the Finn Gen Biobank,^2^ which was released in 2023.

#### 2.1.2 Selection of IVs

In MR analysis, IVs serve as intermediaries between and facilitate the investigation of causal relationships between exposure factors and outcomes. IVs typically comprise genetic variants, with SNPs being the most frequently employed. SNPs associated with Intestinal flora were acquired from the IEU Open GWAS project.^3^ Our focus was specifically on SNPs displaying a strong association with exposure at the genome-wide significance level (*p* < 5 × 10^−8^), but unfortunately, the number of SNPs screened by this condition was very small. To obtain more SNPs to be studied, we used a threshold of *p* < 1 × 10^−5^ for IV screening within a 10,000 kb aggregation window, and demonstrating low linkage disequilibrium (*R*^2^ < 0.001). To satisfy the independence and exclusivity assumptions, we searched the PhenoScannerV2 database^4^ for SNPs strongly associated with intestinal flora factors (e.g., smoking behavior and body mass index) and SNPs associated with the outcome variable (osteoporosis), and no relevant confounding factor SNPs were found (Kamat et al., 2019). Further details are available in Supplementary Table S1. SNPs that showed an association with the outcome at a significance level of *p* < 5 × 10^−5^ were excluded from the analysis. The *F*-statistic was then utilized to validate the strong associations between the independent variables (IVs) and the exposure. In general, associations with F-statistics exceeding 10 were considered to meet the criteria for strong associations (Pierce et al., 2011).

### 2.2 Animals and materials

C57BL/6J female mice (10 weeks old) were obtained from SPF (Beijing) Biotechnology Co., Ltd. [Beijing, China, Certificate No: SCXK (Jing) 2019-0010], and housed in the Experimental Animal Center of Yunnan University of Traditional Chinese Medicine [SYXK (Dian) K2022-0004]. The animals were kept in SPF-grade standard laboratory conditions at 25 ± 2°C, 50 ± 5% humidity, and a 12 h light/dark cycle. Following a 1 week acclimatization period, the mice were randomly divided into two groups (*n* = 5 for each group): a sham surgery group, and an OVX group, which underwent bilateral oophorectomy. After surgery, the mice were provided with sterile distilled water and SPF-grade feed.

After 9 weeks, the mice were anesthetized with isoflurane, and fecal samples were collected and frozen in liquid nitrogen. The samples were then transferred a −80°C ultra-low temperature refrigerator, and femoral, colon, and serum samples were collected for further analysis. This animal experiment was approved by the Experimental Animal Ethics Review Committee of Yunnan University of Traditional Chinese Medicine (No. R-062023004).

We utilized occludin primary antibody, ZO-1 primary antibody, claudin-1 primary antibody (Wuhan Servicebio Technology Co., Ltd., China, GB111401, GB111402, GB12032); CY3-labeled goat anti-rabbit igg secondary antibody (Wuhan Servicebio Technology Co., Ltd., China, GB21303); micro computed tomography (CT) (Bruker, Germany, BrukerSkyScan 1,276); and a small-animal anesthesia machine (Reward Life Science and Technology Co. Ltd., Shenzhen, China, R500) during the experiment.

### 2.3 Detecting bone analysis parameters through micro CT

The fixed femur tissue was positioned on the micro CT carrier stage to scan with the following parameters: 6.5 μm resolution, 350 ms exposure time, 180° scanning angle. The original image was obtained after scanning. The initial images were reconstructed in specific areas using the 3D reconstruction software NRecon. The region of 200 layers beneath the growth plate was analyzed using the CT analyzer. Uniform parameters were set to calculate the total volume of tissue (TV), bone volume (BV), volume ratio (BV/TV), trabecular bone mineral density (Tb. BMD), number of trabeculae (Tb. N), and trabecular separation (Tb. sp).

### 2.4 Observation of microstructural changes in bone and colon tissues using hematoxylin and eosin staining

We used paraffin sections for histological analysis of the femoral and colonic tissues, and all femurs were fixed in 4% paraformaldehyde and decalcified in 10% ethylenediaminetetraacetic acid (EDTA, pH 7.0, Servicebio, China). After paraffin embedding, the samples were cut into 4-μm-thick sections. HE staining was then performed, and the slides scanned using CaseViewer 2.3 to assess the degree of osteoporosis.

### 2.5 Bone turnover and inflammatory indicators

According to the instructions of the kit, the expression levels of LPS, ALP, BGP, TRACP-5b, and CTX-1 in the mouse serum were detected using an enzyme linked immunosorbent assay, and the supernatants of the mouse colon tissue homogenate were extracted to detect the expression levels of IL-6, IL-1β, TNF-α, LPS, and SIgA.

### 2.6 Immunofluorescence detection of gut barrier

After fixation of the colon samples, sequential dehydration, transparency, embedding, sectioning, dewaxing, antigen repair, serum sealing, and addition of occludin, ZO-1, claudin-1 primary antibody, and corresponding secondary antibody was performed. We added 4′,6-diamidino-2-phenylindole (DAPI) to re-stain the cell nuclei, and autofluorescence quencher to quench the autofluorescence of the tissue. The slides were sealed using anti-fluorescence quenching sealer, and image acquisition (excitation wavelength 330–380 nm, emission wavelength 420 nm; CY3 excitation wavelength 510–560 nm, emission wavelength 590 nm) was performed.

### 2.7 16S rRNA measurement of mice gut microbiota

The cetyltrimethylammonium bromide (CTAB) method was chosen for the extraction of total DNA from mouse fecal samples, and the DNA was quantified using an ultra-micro spectrophotometer. The quality of the DNA was detected by agarose gel electrophoresis, using primers 341F (5′-CCTACGGGNGGCWGCAG-3′) 805R (5′-GACTACHVGGGTATCTAATCC-3′) for polymerase chain reaction amplification, and the amplified products were recovered and purified after detection by 2% agarose gel electrophoresis. Sequencing libraries were constructed, and 2 × 250 bp double-end sequencing was performed using a NovaSeq 6000 sequencer to amplify the V3–V4 variable region of bacterial 16S rDNA. Sequencing was performed by Shanghai Biotree Biomedical Biotechnology Co., Ltd.

### 2.8 Statistical analysis

#### 2.8.1 MR analysis

In the two-sample MR analyses, the inverse variance weighting (IVW) model is most capable of detecting causality, so this method was used to determine causal effects (Hartwig et al., 2017). The results of the IVW method were also compared with those of the weighted median, weighted model, and MR Egger methods. The persuasiveness of the findings is enhanced when all four models exhibit consistency. The heterogeneity of the IVW model was evaluated using Cochran’s *Q* test (a *p*-value <0.05 indicates the presence of heterogeneity). It is crucial to acknowledge that the existence of heterogeneity does not necessarily invalidate the IVW model; MR Egger’s approach accommodates a non-zero intercept and can identify multiple validities. Additionally, a leave-one-out analysis was conducted to determine if the exclusion of individual SNPs had a substantial impact on the outcomes. The MR-PRESSO method was used to detect outliers, and, if present, these were removed and analyzed again by MR. Finally, data on positive results found by two-sample MR (TSMR) analysis were collated for reverse TSMR analysis of osteoporosis and gut microbiota to check for reverse causality. All analyses were performed in R software (version 4.2.1) using the TwoSampleMR (version 0.5.8), MendelianRandomization (version 0.8.0), and MRPRESSO package (1.0) (Hemani et al., 2018).

#### 2.8.2 Bioinformatics and statistical analysis

The bipartite data obtained from sequencing were split, spliced, and filtered to obtain high-quality clean labels. Denoising was performed using DADA2 to obtain feature tables and feature sequences. Diversity was calculated by normalizing to the same random sequence. The feature abundance was then normalized using the relative abundance of each sample according to the SILVA (release 138) classifier. Alpha vs. beta diversity was computed by QIIME2, and the R package was plotted. Other experimental results data were analyzed using GraphPad Prism 9 software, a one-way analysis of variance was used for comparisons between multiple groups, and the t-test of two-sample means was used for comparison between two groups. The data are expressed as mean ± SD (standard deviation), and differences were considered statistically significant at *p* < 0.05.

## 3 Results

### 3.1 MR results

#### 3.1.1 Description of IVs selected for inclusion

In total, 2,037 SNPs were chosen as IVs linked to 196 bacterial taxa in the genetic study of gut microbiota and osteoporosis. These SNPs cover 9 phylums (103 SNPs), 16 classes (179 SNPs), 20 orders (217 SNPs), 35 families (339 SNPs), and 131 genera (1,199 SNPs). All IVs had F-statistics exceeding 10, suggesting no bias from weak instrumental variables (refer to Supplementary Table S1 for more information). The MRPRESSO method was employed for subsequent evaluation of multidirectionality, which led to the identification and subsequent removal of the outliers SNPs: rs66710942, rs894996, and rs112893842.

#### 3.1.2 TSMR analysis of gut microbiota in osteoporosis

**Figure 2:**
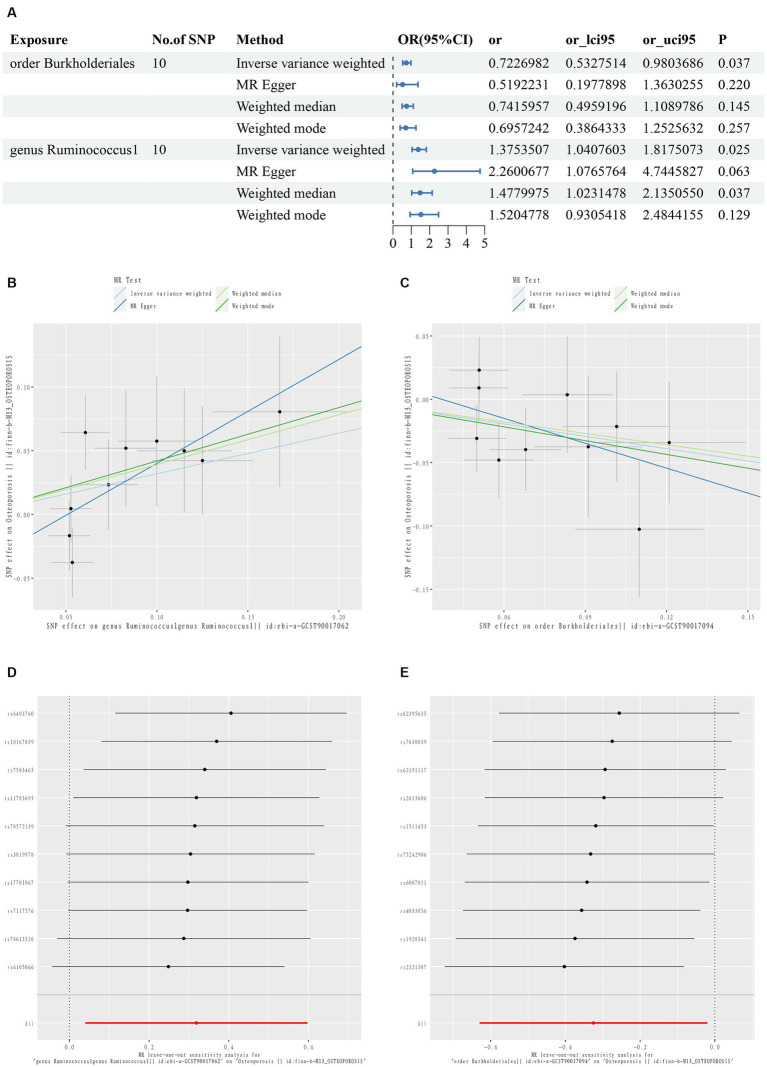
First diagram for the results of TSMR analysis for associations between intestinal flora and osteoporosis **(A)**. The causal effects of *genus Ruminococcus1* on osteoporosis in different MR methods **(B)**. The causal effects of *order Burkholderiales* on osteoporosis in different MR methods. **(C)** Scatter plot. MR leave-one-out sensitive analysis for *genus Ruminococcus1* on osteoporosis **(D)**. MR leave-one-out sensitive analysis for *order Burkholderiales* on osteoporosis **(E)**.

The TSMR results are summarized in Figures 2A–C and Supplementary Table S2. Two taxonomic floras were statistically significant in the MR analysis of osteoporosis, one at the order level and one at the genus level. *g_Ruminococcus1* was a risk factor for osteoporosis, while *O_Burkholderiales* was a protective factor for osteoporosis.

The IVW results showed that *g_Ruminococcus1* [OR = 1.375; 95% confidence interval (CI): 1.041–1.818] was positively correlated with the risk of osteoporosis, and *O_Burkholderiales* (OR = 0.723; 95% CI: 0.533–0.980) was negatively correlated. A heterogeneity analysis of these flora revealed no heterogeneity arising (*p* > 0.05). The MR Egger analysis found no horizontal pleiotropy between *g_Ruminococcus1* (*p* = 0.194), *O_Burkholderiales* (*p* = 0.259), and outcome (*p* > 0.05). Further assessment of pleiotropy using the MRPRESSO method again revealed no outliers, indicating that the findings of the IVW method were reliable (Supplementary Table S3).

The MR results and sensitivity analyses with genome-wide significant differences are described in Supplementary Tables S4, S5. The leave-one-out method sensitivity analyses also showed no significant differences in the estimation of the pathogenic effects of *g_Ruminococcus1* and *O_Burkholderiales* on osteoporosis, regardless of the exclusion of the IVs, indicating the robustness of the MR analyses (Figures 2D,E).

#### 3.1.3 Reverse MR analysis

To avoid reverse causality from affecting the above findings, we conducted a reverse MR analysis with osteoporosis as the exposure and the positive results for gut microbiota as the outcome. Instrumental variables were extracted in the (*p* < 5 × 10^−8^) conditions, resulting in no eligible SNPs, there was no indication of a causal effect of osteoporosis on the *g_Ruminococcus1* and *O_Burkholderiales*.

### 3.2 Successful replication of the osteoporosis model in mice

**Figure 3:**
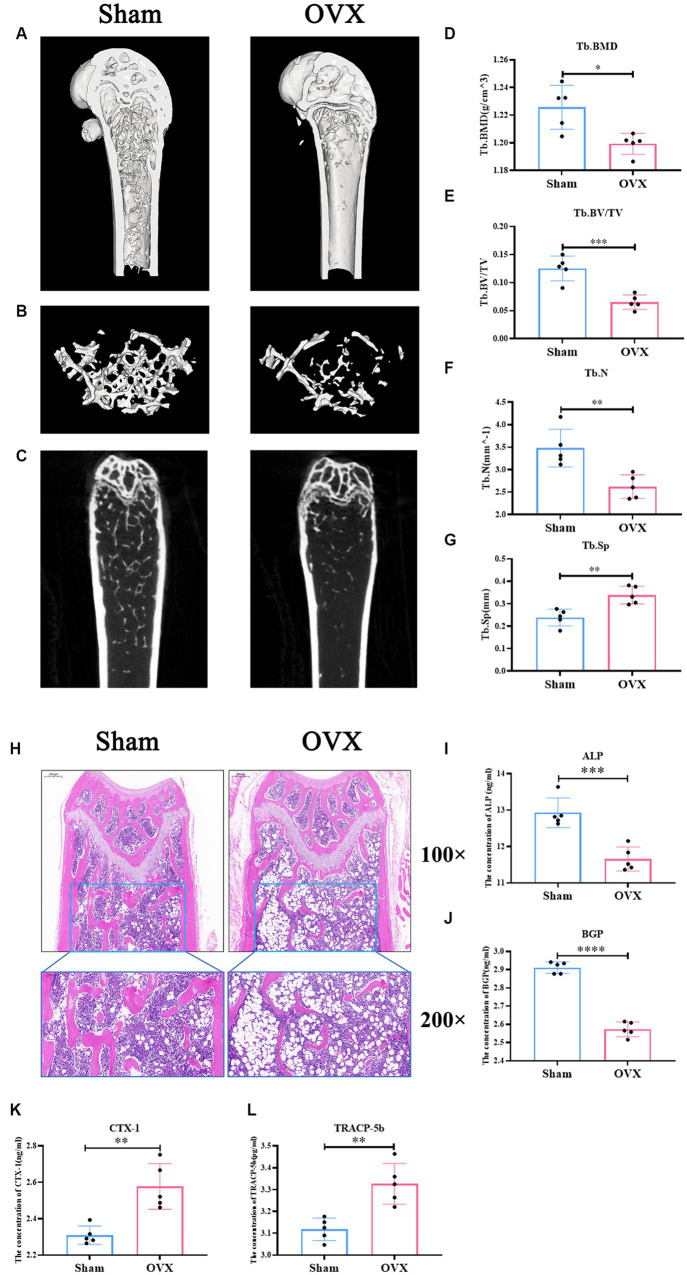
Successful replication of a mouse model of osteoporosis. Sagittal longitudinal 3D view of the distal femur **(A)**; 3D view of the bone trabeculae **(B)**; coronal longitudinal plan view of the distal femur **(C)**; bone trabecular density **(D)**; bone volume fraction **(E)**; bone trabecular number **(F)**; bone trabecular separation **(G)**. Distal femur tissue stained with hematoxylin and eosin 100×, 200× **(H)**. The expression level of alkaline phosphatase in serum (ALP, **I**); the expression level of bone guardian protein in serum (BGP, **J**); the expression level of Collagen type I C-terminal peptide in serum (CTX-1, **K**), the expression level of antitartrate acid phosphatase-5b in serum (TRACP-5b, **L**). Data are expressed as mean ± SD (*n* = 5). One-way ANOVA procedure followed by Tukey test was used to evaluate the statistical significance, ^*^p < 0.05 and ^**^p < 0.01.

Micro CT data (Figures 3A–G) showed that femoral Tb. BMD, BV/TV, Tb. N, and Tb. sp were significantly lower in the OVX group of mice compared with the sham group (*p* < 0.05), suggesting that significant bone loss occurred in the OVX mice. Changes in the bone microstructure were apparent in the femurs in the two groups of mice (Figure 3H). Compared with the sham group, the trabecular meshwork of the femurs of mice in the OVX group was disrupted, the thickness of the trabeculae was thinned, some of the trabeculae appeared to be broken, and there was an increase in the formation of fat vacuoles, suggesting a deterioration of the bone microstructure in the OVX mice. Regarding bone transformation levels of mice in both groups (Figures 3I–L), compared with the sham group, the expression levels of bone formation factors (ALP, BGP) in the serum of mice in the OVX group decreased (*p* < 0.001), and the expression levels of bone resorption factors (TRACP-5b, CTX-1) increased (*p* < 0.001), with bone resorption being greater than bone formation, resulting in significant bone loss. Therefore, the replication of the osteoporosis mouse model was successful.

### 3.3 High-throughput sequencing of 16S rDNA

#### 3.3.1 Venn and α-diversity analysis of gut microbiota

**Figure 4:**
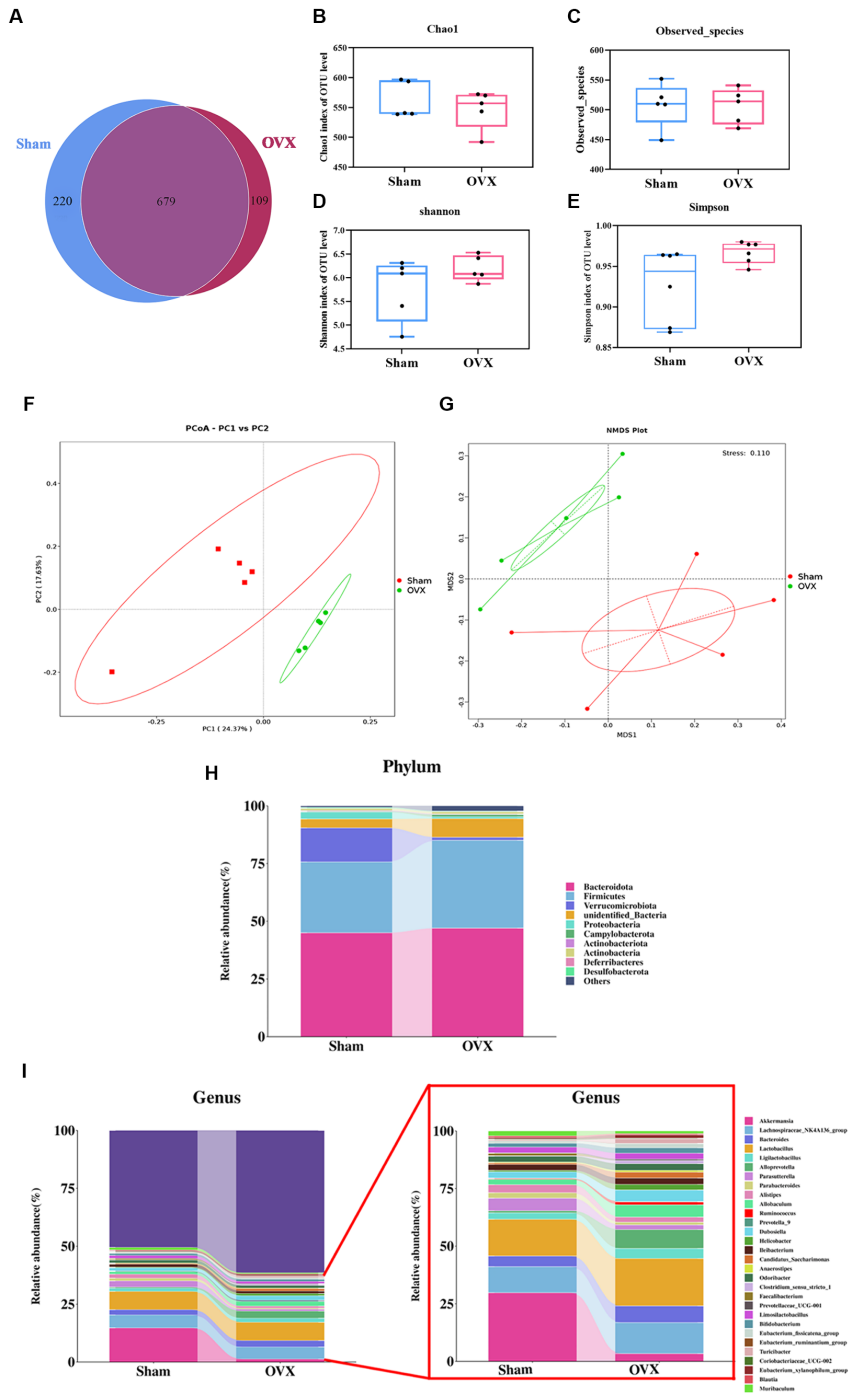
Diversity and abundance analysis of microorganisms. Venn diagram of the two groups of OTUs **(A)**; Chao 1 index of α-diversity **(B)**; observed species index of α-diversity **(C)**; Shannon index of α-diversity **(D)**; Simpson index of α-diversity **(E)**; PCoA plot of β-diversity in two groups **(F)**; NMDS plot of β-diversity in two groups **(G)**. Histogram of species distribution of the intestinal flora at the phylum level **(H)**; histogram of species distribution of the intestinal flora at the genus level **(I)**. Data are expressed as mean ± SD (*n* = 5).

A Venn analysis showed a total of 679 operational taxonomic units (OTUs) in the two groups (Figure 4A), of which each group had unique OTUs, and the OTUs in the OVX group showed a decreasing trend compared with the sham group. Chao1 index and observed species showed a decreasing trend in the total number of species in the feces of the mice in the OVX group compared with the sham group, indicated in the samples of gut microbiota of osteoporotic mice after depopulation. The total number of species detected showed a decreasing trend (Figures 4B,C). Shannon index and Simpson showed a high trend in the OVX group compared to the sham group, indicating a more even distribution of community species in the OVX group (Figures 4D,E).

#### 3.3.2 β-diversity analysis of the gut microbiota

The principal component analysis results (Figure 4F) showed that the first principal component could explain 24.37% of the differences between groups, and the second principal component could explain 17.63% of the differences between groups. Non-metric multidimensional scaling analysis (Figure 4G) showed that the Stress value was 0.110, indicating that the model was less different from the original data and that the model was acceptable. An analysis of similarity between groups (ANOSIM) showed that *R* > 0 and *p* < 0.05 when groups were compared two by two, indicating that the differences between groups were obvious and the results of the differences were significant. This indicates that the community structure was more different between groups and the grouping was effective and scientific.

#### 3.3.3 Structural abundance analysis of the gut microbiota

The dominant bacteria in each group of samples at the phylum level are roughly the same (Figure 4H), mainly concentrated in the four major phyla of *Bacteroidetes*, *Firmicutes*, *Verrucomycota*, and *Proteobacteria*, but with differences in relative abundance. Compared with the sham group, the OVX group showed an increase in the phyla *Bacteroidetes*, *Firmicutes*, and *Campylobacter* in the feces of mice, while the phyla *Verrucomycota*, *Proteobacteria*, and *Actinobacteria* decreased. At the genus level (Figure 4I), the samples from each group were mainly dominated by the genera *g_Akkermansia, g_Parabacteroides, g_Lactobacillus, g_Prevotella, g_Alistipes, g_Ruminalococcus,* etc. Compared with the sham group, the feces of mice in the OVX group showed an increase in *g_Bacteroidetes, g_Lactobacillus*, and *g_Ruminalococcus*, and a decrease in the genera of *g_Akkermansia, g_Prevotella, g_Parabacteroides, and g_Alistipes*.

#### 3.3.4 LEfSe multilevel species difference discriminant analysis

**Figure 5:**
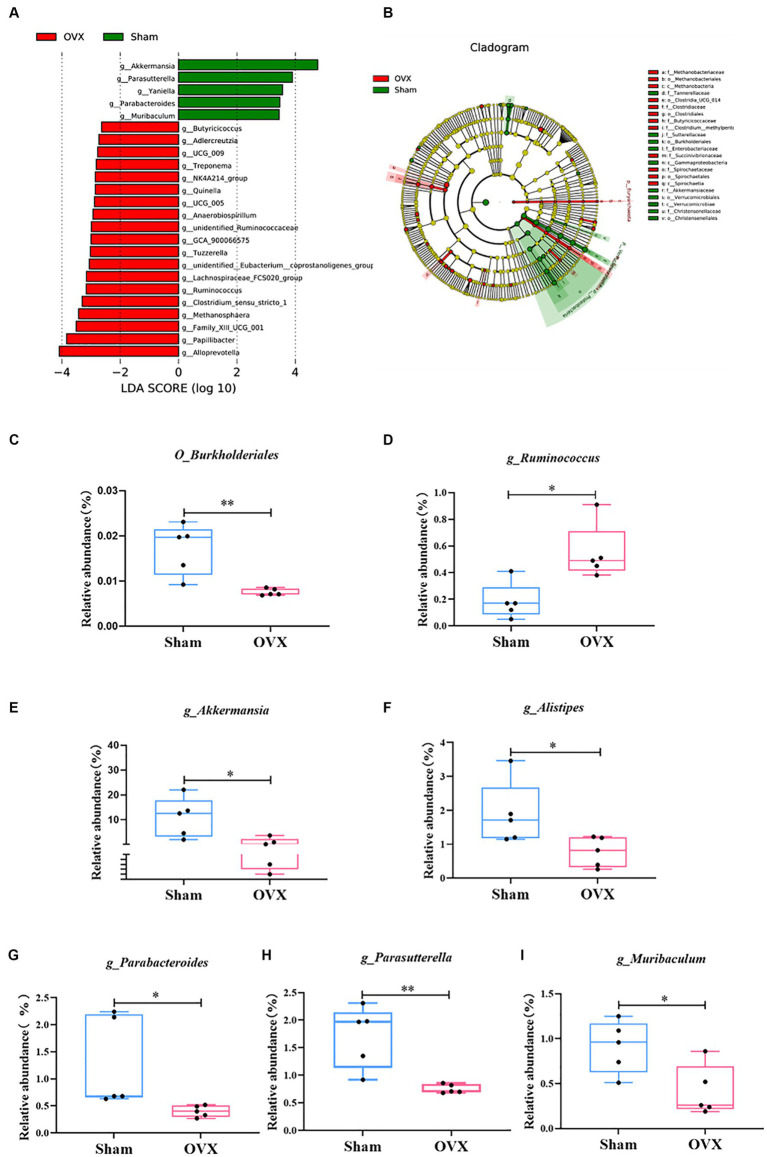
LEfSe multilevel discriminant analysis of species differences. LEfSe analysis histogram **(A)**; LEfSe analysis evolutionary branching diagram **(B)**; *O_Burkjolderiales* **(C)**; *g_Ruminococcus* **(D)**; *g_Akkermansia* **(E)**; *g_Alistipes* **(F)**; *g_Parabacteroides* **(G)**; *g_Parasutterella* **(H)**; *g_Muribaculum* **(I)**. Data are expressed as mean ± SD (*n* = 5). One-way ANOVA procedure followed by Tukey test was used to evaluate the statistical significance, ^*^p < 0.05 and ^**^p < 0.01.

An LEfSe analysis (Figure 5) was used to screen for significantly different groups of bacteria at the genus level in each group of mice with a linear discriminant analysis value of >4. A statistical analysis revealed that the different genera in the sham group were *O_Burkholderiales, g_Akkermansia, g_Alistipes, g_Parabacteroides, g_Parasutterella*, and *g_Muribaculum* (^*^p < 0.05 and ^**^p < 0.01); the differential genus in the OVX group was *g_Ruminococcus* (^*^p < 0.05). Due to database updates and renaming of the bacterial genus, the current version of SILVA database cannot find *g_Ruminococcus 1*. By tracing the original data and annotating literature, comparing various versions of the SILVA database (Supplementary Table S6 and www.arb-silva.de/treeviewer), we found that the current *g_Ruminococcus* and *g_Ruminococcus 1* is consistent at the genus level, which validated that *g_Ruminococcus* can represent *g_Ruminococcus 1*; its abundance expression level can reflect the *g_Ruminococcus 1* validation results (Henderson et al., 2019; Kurilshikov et al., 2021).

### 3.4 Colon injury detection

#### 3.4.1 HE staining of the colon and detection of inflammatory factors

**Figure 6:**
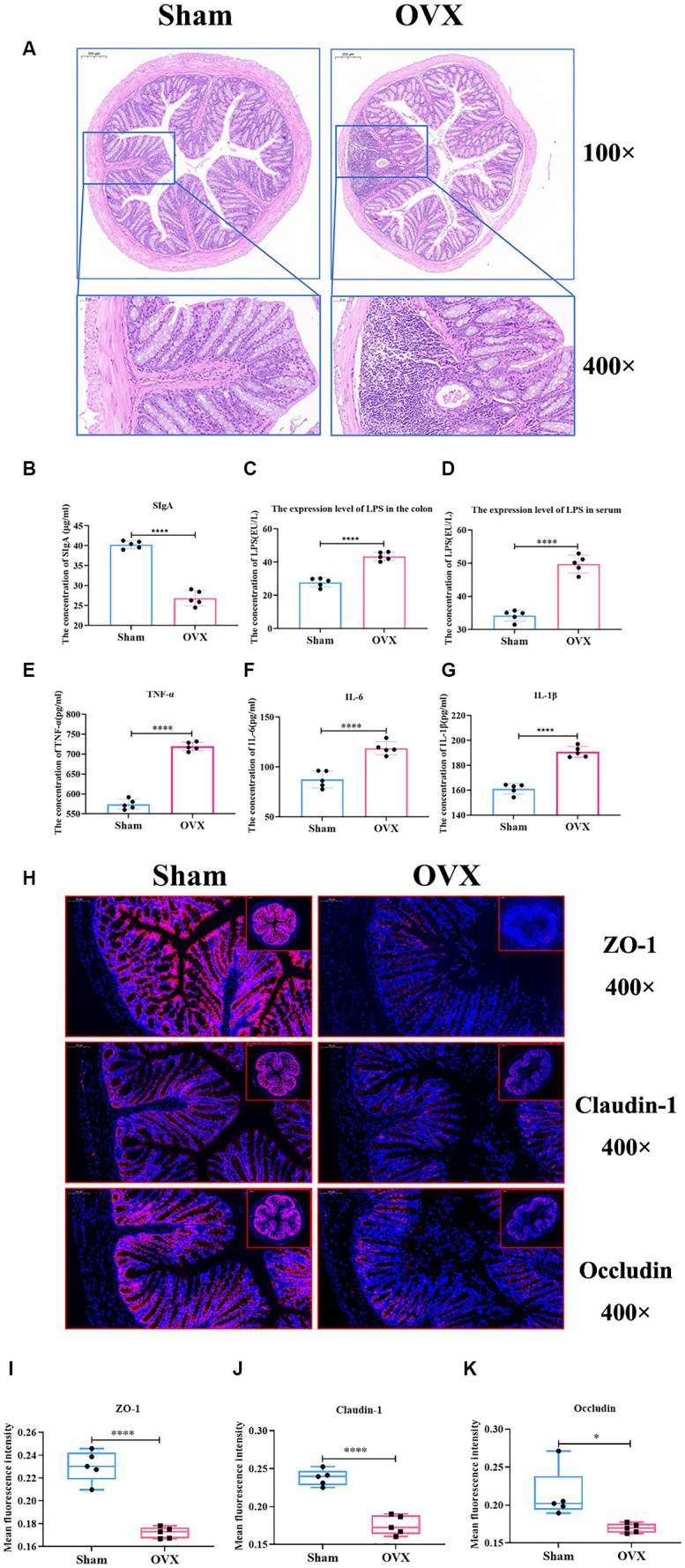
Detection of inflammatory factors and barrier indicators in colon tissue. Colon HE staining of two groups of mice **(A)**; the expression level of colon SIgA **(B)**; the expression level of LPS in the colon **(C)**; the expression level of LPS in serum **(D)**; the expression levels of TNF-α, IL-6 and IL-1β in the colonic tissues **(E–G)**; immunofluorescence detection of colon barrier indicators ZO-1, claudin-1, and occludin and their relative fluorescence expression levels **(H–K)**.

Compared with the sham group, the OVX group showed superficial epithelial damage of the colonic mucosa, decreased cup cells, shallow and irregularly arranged intestinal crypts, and a large number of inflammatory cells infiltrated locally (Figure 6A). Compared with the sham group, the expression levels of SIgA, IL-6, IL-1β, and TNF-α in the colon of the OVX group were significantly lower (*p* < 0.001), and LPS in the serum and colon tissues were significantly higher (*p* < 0.001), which indicated that, after OVX, the mucosal immunity of colon tissues was reduced and the expression levels of inflammatory factors were elevated, and that the entry of LPS into the bloodstream from the damaged gut barrier was causing systemic inflammation (Figures 6B–G).

#### 3.4.2 Immunofluorescence detection of gut barrier indicators

Compared with the sham group, the expression of intestinal tight junction proteins ZO-1, claudin-1, and occludin was significantly reduced in the OVX group (*p* < 0.01) (Figures 6H–K), showing that the integrity of the gut barrier of osteoporosis mice induced by OVX was damaged.

### 3.5 Correlation analysis between microbial communities and environmental factors

**Figure 7:**
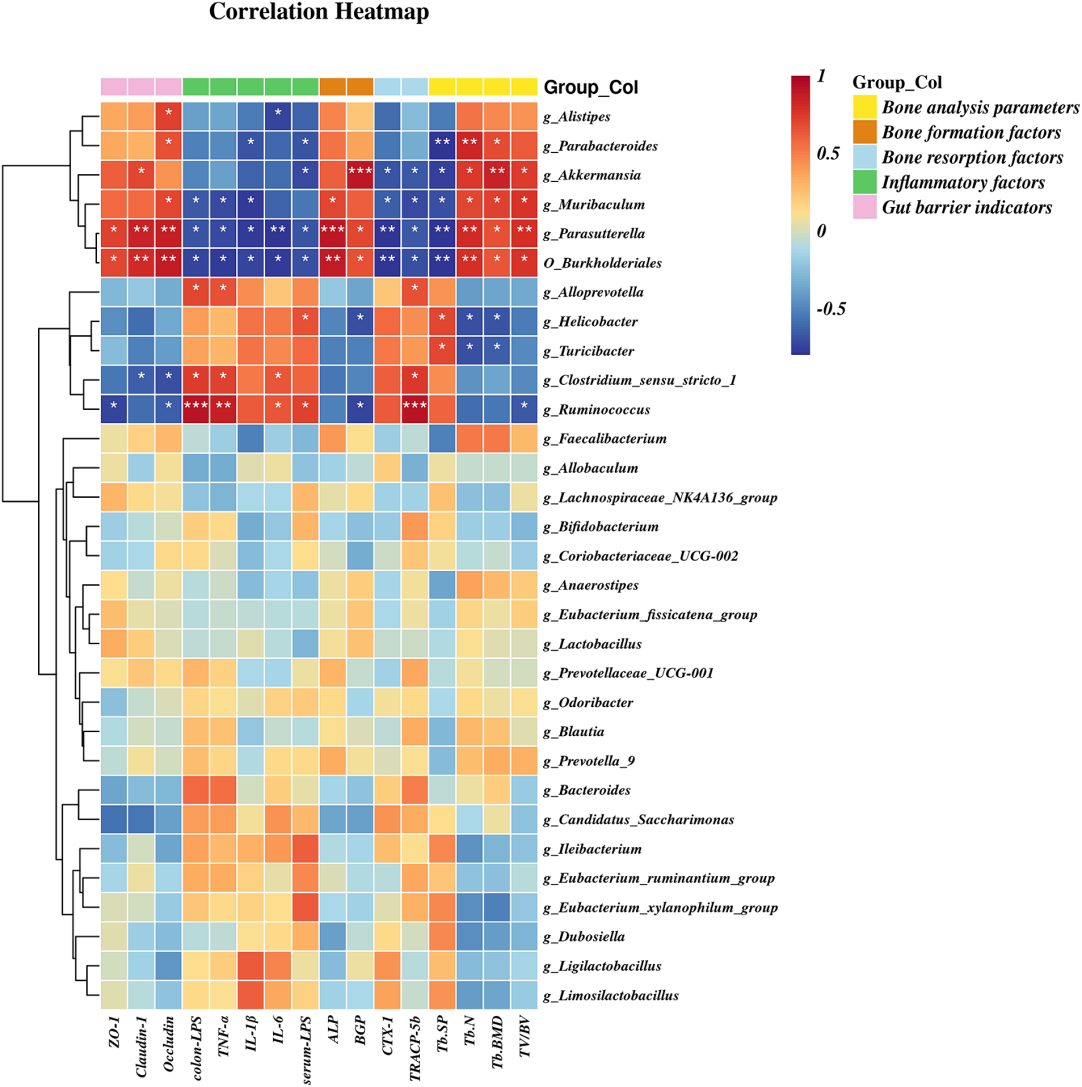
Heat map of the correlation between differential microbiota and cytokines (^*^p < 0.05, ^**^p < 0.01, and ^***^p < 0.001).

**Figure 8:**
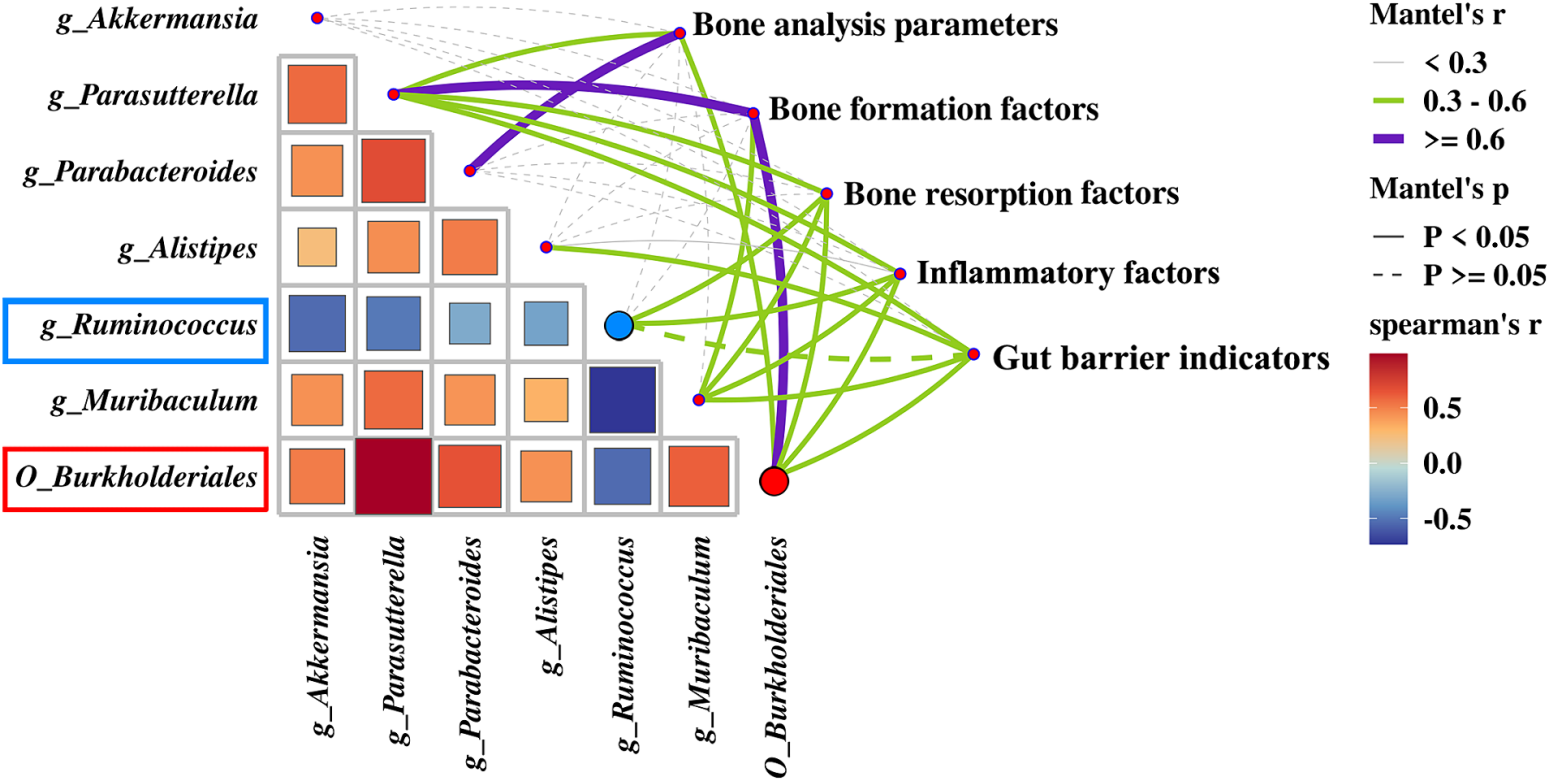
Mantel test of between 7 differential bacteria and bone analysis parameters, bone formation factors, bone resorption factors, inflammatory factors, and gut barrier indicators.

**Figure 9:**
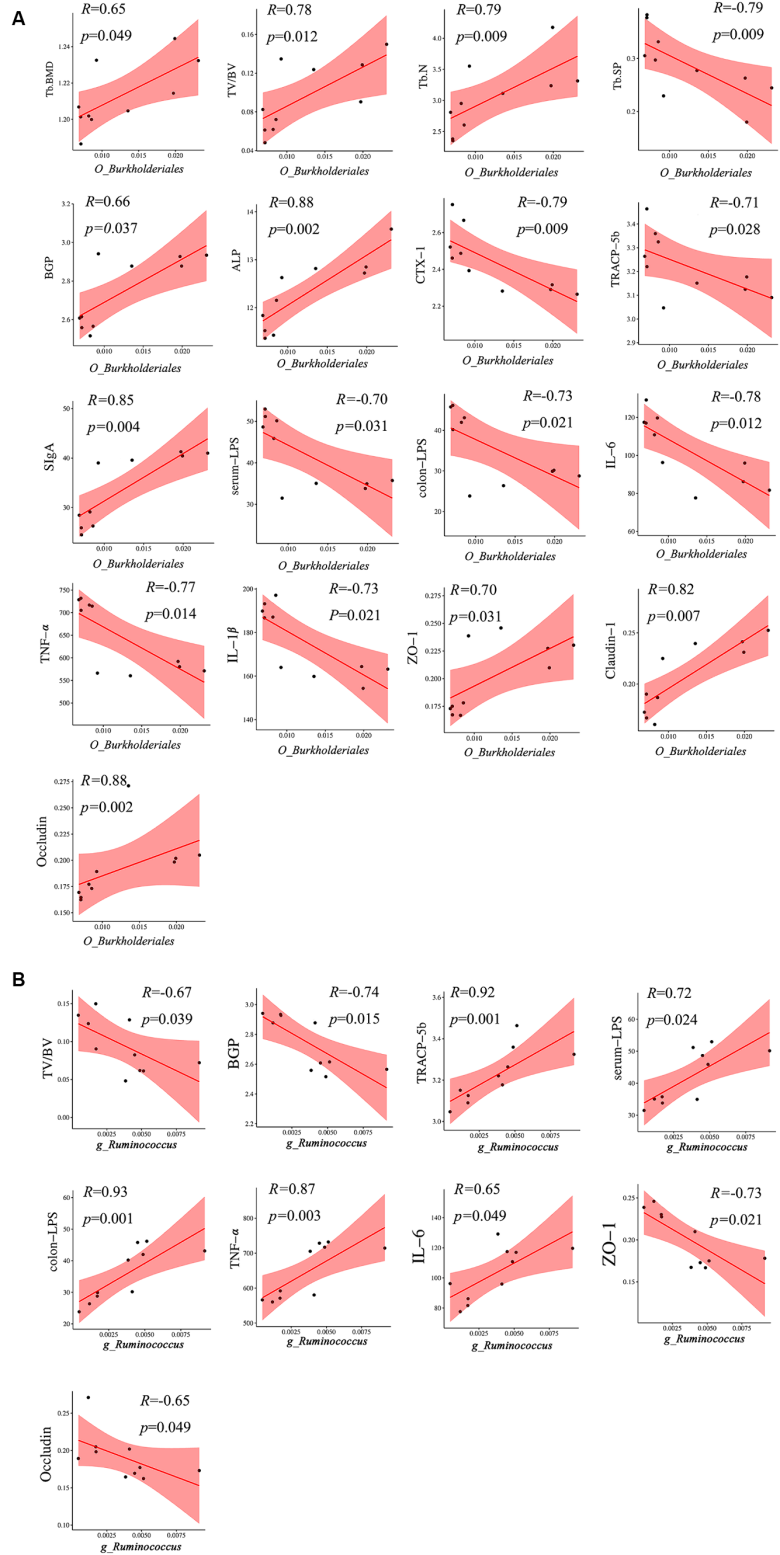
Scatter plot of the correlation between *g_Ruminococcus* and *O_Burkjoldriales* and environmental factors. Spearman’s correlation analysis, *r* > 0.6, *p* < 0.05.

A heatmap correlation analysis between *O_Burkholderiales* and the top 30 genera ranked bacteria with environmental factors was used (Figure 7). Based on whether the data conforms to a normal distribution, a Spearman correlation analysis was selected to screen out seven bacteria with significant differences in bone analysis parameters, bone formation factors, bone resorption factors, colitis factors, and colon barrier proteins for a Mantel test (Figure 8). According to *r* > 0.6 and *p* < 0.05, the correlation scatter plot of *O_Burkholderiales* and *g_Ruminococcus* (Figure 9) was performed, and we found that the abundance of g_*Ruminococcus* was strongly positively correlated with the expression levels of IL-6, TNF-α, LPS, and TRACP-5b, and strongly negatively correlated with BV/TV, BGP, ZO-1, and occludin. The abundance of *O_Burkholderiales* was strongly negatively correlated with the expression levels of inflammatory factors and bone resorption factors, and strongly positively correlated with bone analysis parameters, bone formation factors, and gut barrier indicators.

## 4 Discussion

Our study provides evidence for a causal relationship between gut microbiota and osteoporosis. Through MR analysis we found that *g_Ruminococcus* was a risk factor for increased risk of osteoporosis, while *O_Burkholderiales* was a protective factor for decreased risk of osteoporosis; however, as a limitation of MR analysis, our study was conducted in a European population. This limits the generalizability of our research findings to other racial or ethnic groups. The effect of the gut microbiota on osteoporosis in different subgroups (e.g., age, sex, place of birth, diet) of the population could not be analyzed, and there may be a small overlap in the cohorts used to determine exposures and outcomes, but the F-statistic for IV is sufficient to prevent bias due to weak instrumental variables.

In the validation of the animal experiments, we found a trend toward lower gut microbial abundance and diversity in osteoporotic mice; *g_Ruminococcus* was highly and statistically significantly expressed in the osteoporosis model mice, and *O_Burkholderiales* was significantly elevated in the sham-operated control group. Moreover, there was obvious gut barrier damage in the osteoporotic mice, with decreased expression of SIgA, increased expression of LPS, IL-6, IL-1β, and TNF-α in intestinal tissues, as well as increased serum levels of LPS, suggesting that the intestinal microenvironment of the mice was disturbed in the osteoporosis mouse model, leading to damage of the gut barrier. This induced intestinal inflammation and translocation of LPS into the bloodstream, and then caused systemic chronic inflammation.

*g_Ruminococcus* is an anaerobic bacterium that plays different roles in various diseases. Although *g_Ruminococcus* can ferment complex sugars and produce short-chain fatty acids (SCFAs), not all *g_Ruminococcus* bacterium are beneficial to health, and an overabundance of certain strains can cause diseases (Crost et al., 2023). The expression of *g_Ruminococcus* is decreased in cerebral palsy and infantile allergy patients (Zheng et al., 2016; Huang et al., 2019), while its abundance is significantly increased in some digestive diseases (e.g., Crohn’s disease, inflammatory bowel disease, irritable bowel syndrome, etc.), spondylarthritis, and asthma (Henke et al., 2019). Patients with inflammatory bowel disease experience transient and intense *g_Ruminococcus* bloom, positively correlated with the disease (Hall et al., 2017). Whereas inflammatory bowel disease exacerbates bone mineral loss and causes an imbalance between osteoblasts and osteoclasts, leading to an increased incidence of osteoporosis in patients with inflammatory bowel disease (Adriani et al., 2014; Dai et al., 2023), it has also been shown that *g_Ruminococcus* bacteria in the intestinal tract of patients with osteoarthritis of the spine are positively correlated with the history of inflammatory bowel disease activity (Vereecke and Elewaut, 2017), suggesting that the role of *g_Ruminococcus* bacteria in bone metabolic abnormalities and intestinal inflammation do correlate. We found that *g_Ruminococcus* has a strong positive correlation with the expression levels of colon-LPS, TNF, and TRACP-5b, while it has a strong negative correlation with BV/TV, BGP, ZO-1, and occludin. However, as a gram-positive bacterium, *g_Ruminococcus* does not produce LPS and may work in conjunction with other bacteria to promote an increase in LPS and exacerbate gut barrier damage.

Despite the relatively low abundance of *Burkholderiales* in the human gut, we found a strong negative correlation between its abundance level and Tb. sp and CTX-1, with a strong positive correlation with Tb. N, ALP, claudin-1, and occludin. This suggests that *Burkholderiales* can repair damage to the gut barrier, promote bone formation, and increase bone density, and that *Burkholderiales* itself has a unique ability to degrade a variety of compounds (including exogenous substances), and its effect on bone may play an indirect osteoprotective role by eliminating potential pathogenic cytokines in the gut, enriching the diversity of the intestinal microbiota in conjunction with other bacterial flora (Wicaksono et al., 2023), and maintaining the integrity of the intestinal tract (Luo et al., 2023). Meanwhile, this study also identified *g_Akkermansia, g_Parabacteroides, g_Alistipes, g_Parasutterella,* and *g_Muribaculum* as possible differential genera affecting BMD, among which *B. Akkermansia* is a potential probiotic located in the mucus layer of the intestine, which produces a variety of mucin-degrading bio-enzymes and ferments to produce SCFAs (Collado et al., 2007). This is important for the alleviation of inflammatory bowel disease, preventing obesity, diabetes, and cardiovascular metabolic diseases (Cani et al., 2022). Liu et al. (2021) found that *Akkermansia muciniphila* could play a role in promoting bone formation and inhibiting bone resorption by releasing extracellular vesicles into the bone tissue to alleviate bone loss in postmenopausal osteoporotic mice. This experiment found that the abundance of *g_Akkermansia* has a strong positive correlation with bone formation factors and Tb. BMD, indicating that it may be a protective factor promoting bone formation. *Alistipes* is a gram-negative bacterium belonging to the phylum Bacteroidetes, and its main metabolites are succinic acid, acetic acid, and propionic acid (Parker et al., 2020). An MR study on the causal relationship between the gut microbiota and bone mineral density (BMD) showed that *Alistipes* have a protective effect on BMD (Wang et al., 2023). Furthermore, the relative abundance of *Alistipes* is decreased in osteoporosis patients (Huang et al., 2022); however, we found that the expression abundance of *Alistipes* in OVX mice was significantly reduced, showing a strong positive correlation with gut barrier indicators, indicating that *Alistipes* may also be a potential mediator to regulate the microbiota-gut-bone axis.

*Parasutterella* is a member of the core microbiome of healthy feces in the human gastrointestinal tract and is one of the most reported taxa in the Betaproteobacteria class in the intestine. This bacterium can stably colonize the mouse intestine without causing immune reactions or fluctuations in gut microbiota composition (Ju et al., 2019). Several animal models and human studies have shown that the relative abundance of *Parasutterella* is closely related to the health outcomes of different hosts, such as inflammatory bowel disease, obesity, and diabetes etc. (Henneke et al., 2022). In our experiment, there was a strong positive correlation between its expression abundance and serum bone formation factors (ALP, BGP), indicating that changes in its abundance may have a positive regulatory effect on bone formation.

*Parabacteroides*, a gram-negative anaerobic bacterium, is commonly colonized in the gastrointestinal tract of many species, serving as a core component of the human and mouse gut microbiota. *Parabacteroides* is strongly associated with various health outcomes, synthesizes succinate, participates in the maintenance of bile acid homeostasis and cholesterol metabolism, and its levels correlate significantly and negatively with disease states such as obesity, NAFLD, and diabetes. Furthermore, it exerts a positive modulatory role in glucose-lipid metabolism (Ezeji et al., 2021; Cui et al., 2022). We found a strong positive correlation between its expression abundance and Tb. BMD and Tb. N, indicating that changes in its abundance may have a positive regulatory effect on bone formation.

*Muribaculum* is an anaerobic strain that can be extracted from conventionally reared C57BL/6J mouse fecal pellets and is a metabolite that can influence T cell populations for immunomodulatory effects (Bang et al., 2023). We found that the abundance of *g_Muribaculum* is strongly negatively correlated with intestinal inflammatory factors and bone resorption factors, and strongly positively correlated with gut barrier proteins and bone formation factors, indicating that *g_Muribaculum* is also a potential bacterium that acts on the microbiota-gut-bone axis to alleviate osteoporosis. Except for *g_Akkermansia*, which acts directly on bone metabolism, several of the above flora have not yet been studied to show their direct effects on bone health, which is expected to be verified in future experiments.

A connection between gut bacteria and BMD in mice has been found (Chen et al., 2023). Germ-free mice tend to have higher bone mass compared to those raised under conventional conditions. Additionally, when fecal transplants from conventional mice are administered, germ-free mice show a decrease in bone mass (Sjogren et al., 2012). This trend is also observed in germ-free mice after drug castration, suggesting that gut bacteria may play a role in bone growth and development through certain mechanisms (Li et al., 2016; Tyagi, 2023); however, the differences in regulation may be closely related to differences in mouse gender, mouse species, cage conditions, and feed used. This relationship between gut microbes and bone metabolism is known as the microbiota-gut-bone axis or gut-bone axis (Tu et al., 2021). Its mechanism of action can directly participate in the control of bone metabolism by regulating the hormone level *in vivo* and the host immune system. It can also indirectly participate in the regulation of bone metabolism by mediating various endogenous metabolites (such as short chain fatty acids, gut derived serotonin, bioactive peptides) or affecting the intestinal mucosal barrier, and the integrity of the intestinal mucosal barrier is an important bridge mediating the microbe gut bone axis (Cooney et al., 2020; Tu et al., 2023; Zhang et al., 2024). The gut barrier is crucial to maintain the equilibrium between the gut bacteria and the host, serving as a protective mechanism against harmful microorganisms (Allaire et al., 2019). Disruption of the gut microbiota can compromise the gut barrier, leading to the release of microorganisms, LPS, and pro-inflammatory cytokines into the bloodstream, causing bacterial translocation and systemic chronic inflammation. This, in turn, affects the bone microenvironment and bone metabolism in the distal bone tissues (Ohlsson and Sjogren, 2015; Shieh et al., 2020). Therefore, impairment of the gut barrier is a critical factor in the microbiota-gut-bone axis. Our study indicates that, in the development process of osteoporosis, the expression abundance of *g_Ruminococcus* is strongly positively correlated with bone loss and gut barrier injury. The expression abundance of *O_Burkholderiales* is strongly negatively correlated with bone loss and gut barrier injury, which also provides a basis for the microbiota-gut-bone axis theory. This also supports the theory of the microbiota-gut-bone axis and the results of the previous systematic evaluation of the association between the gut microbiota and osteoporosis. Interfering with the abundance of *g_Ruminococcus* and *O_Burkholderiales* may affect the bone density or the integrity of the gut barrier and provide a potential biomarker for the diagnosis of osteoporosis.

## Data availability statement

The original contributions presented in the study are publicly available. This data can be found here: https://submit.ncbi.nlm.nih.gov/subs/sra/SUB14154051/overview; PRJNA1065495.

## Ethics statement

The animal study was approved by the Experimental Animal Ethics Review Committee of Yunnan University of Traditional Chinese Medicine and Yunnan University of Chinese Medicine. The study was conducted in accordance with the local legislation and institutional requirements.

## Author contributions

NL: Conceptualization, Data curation, Formal analysis, Validation, Visualization, Writing – original draft. HW: Data curation, Software, Visualization, Writing – original draft. HP: Data curation, Validation, Visualization, Writing – original draft. YW: Data curation, Validation, Writing – original draft. LeL: Data curation, Validation, Writing – original draft. YR: Data curation, Validation, Writing – original draft. SW: Data curation, Validation, Writing – original draft. YM: Validation, Writing – review & editing. ML: Validation, Writing – original draft. JY: Conceptualization, Funding acquisition, Project administration, Resources, Supervision, Writing – review & editing. LvL: Conceptualization, Funding acquisition, Project administration, Resources, Supervision, Writing – review & editing. DQ: Project administration, Resources, Supervision, Writing – review & editing, Conceptualization, Funding acquisition.

## Footnotes

^1^ https://MiBioGen.gcc.rug.nl

^2^ https://r5.finngen.fi

^3^ https://gwas.mrcieu.ac.uk/

^4^ http://www.phenoscannermedschl.cam.ac.uk/
